# Healing the Broken Heart: A Cardiac Odyssey in the Face of Obstetric Crisis

**DOI:** 10.7759/cureus.69534

**Published:** 2024-09-16

**Authors:** Praneeth Ulavala, Hemalatha Bhoompally, Abhishek Vadher, Hari Chandana Kalangi, Tejaswini Muni

**Affiliations:** 1 General Medicine, Narayana Medical College and Hospital, Nellore, IND; 2 Intensive Care Unit, Yashoda Hospitals, Secunderabad, IND; 3 Internal Medicine, Garden City Hospital, Garden City, USA; 4 General Medicine, Kamineni Academy of Medical Scieneces and Research Centre, Hyderabad, IND; 5 Intensive Care Unit, Yasodha Hospitals, Secunderabad, IND

**Keywords:** intra-aortic balloon pump, maternal morbidity, obstetric emergencies, postpartum hemorrhage, takotsubo cardiomyopathy

## Abstract

Postpartum hemorrhage (PPH) is one of the leading causes of maternal morbidity and mortality worldwide. The management of severe PPH remains challenging despite advances in obstetric care. Conventional methods of management with uterotonic agents, surgical techniques, and blood transfusions often fail to provide adequate hemostasis, more so in refractory cases. The intra-aortic balloon pump (IABP) has been recently described as a new therapeutic modality in refractory PPH and is commonly used in cardiology. This case report presents a rare case of severe uncontrollable PPH complicated by Takotsubo cardiomyopathy (TCM). She was managed with the conventional methods by adding a relatively new technique, the IABP. The case thus brings out the potential role of IABP in the management of severe PPH and also reinforces the multidisciplinary approach in obstetrical emergencies.

## Introduction

Postpartum hemorrhage (PPH) is a leading cause of maternal death and morbidity throughout the world. Severe PPH, defined as postpartum blood loss greater than 500 mL after vaginal delivery or more than 1000 mL following cesarean section, is an integrated and critical component requiring immediate intervention to avoid irreversible consequences [1]. However, traditional methods (uterine massage, uterotonic agents, and surgical techniques) can be frequently applied for its management. There is a subset of PPH whose cases do not respond to conservative approaches and require an alternative way to achieve hemostasis as well as stabilization of the homeostatic status. In recent years, the intra-aortic balloon pump (IABP) has been introduced as a rescue therapy for cases of refractory PPH, using an uncommon strategy to enhance tissue perfusion and prevent adverse outcomes [2]. Here, we describe a case of successful management with IABP in addition to supportive measures that have not been previously reported.

Conventional treatment of PPH traditionally consists of initial utero-tonic drugs, massaging the uterus, or manual removal of placental tissue. Further interventions like blood transfusion, surgery (ultrasound-guided procedures or laparoscopy/laparotomy for bimanual compression and evacuation, uterine compression sutures, and hysterectomy), and uterine artery embolization (UAE) may be needed to achieve hemostasis in extreme cases and avoid further complications [3,4].

The role of IABP in PPH

The indication for using an IABP as a last resort to control refractory massive PPH is based on its ability to increase cardiac output and augment tissue perfusion [2]. The use of IABP can serve as an adjunct to the management of bleeding and stabilization in maternal hemodynamics by increasing aortic pressure and decreasing uterine blood flow, which will help avoid consequences associated with severe hemorrhage [5].

Advantages and disadvantages of IABP in PPH

Although the case series could suggest a potential role for IABP in managing refractory PPH, it is essential to weigh the benefits versus limitations. One of the significant advantages of IABP is its reversible nature and rapid onset of action that enables fast hemodynamic support in severely ill patients. Moreover, IABP can be rapidly deployed at the bedside without specialized equipment or extensive training, making it an option widely available for use during emergencies. Nonetheless, it is crucial to recognize the consequences of IABP, such as vascular complications and device-related malfunction leading to hemodynamic collapse. Additionally, the utility of IABP may differ based on the type and depth of PPH, indicating that an individualized patient evaluation in treatment should be considered [6].

Takotsubo cardiomyopathy (TCM) in PPH

TCM, also called stress-induced cardiomyopathy or “broken heart syndrome,” can occur in PPH cases. This condition is triggered by physical stress, leading to temporary left ventricular dysfunction and apical ballooning appearance on cardiac scans. The exact mechanisms behind TCM in PPH have yet to be entirely understood. It is thought to be associated with catecholamine surges, as well as myocardial stunning and microvascular malfunction. For severe cases, management usually consists of providing supportive care using inotropic drugs or mechanical circulatory support devices such as an IABP [7].

## Case presentation

On September 1, 2023, a 22-year-old female presented in the emergency room (ER) with a history of lower segment cesarean section (LSCS) and bilateral tubectomy under spinal anesthesia. She had continuous per vaginal bleeding every fortnight following the procedure, necessitating several admissions for severe per vaginal bleeding. She was admitted to an external hospital on January 30, 2023, because of severe vaginal bleeding, and she was discharged after four to five days. However, on March 8, 2023, her symptoms returned, accompanied by fever and painful urination. She was diagnosed with secondary PPH with severe anemia and hemorrhagic shock when she was taken to a local hospital on March 11, 2023. A computed tomography (CECT) scan of the abdomen showed endometrial and endocervical hematomas. After being treated intensively with dual inotropes as well as numerous blood and blood products transfusions, including four units of packed red blood cells and four units of fresh frozen plasmas, she continued to have continuous per vaginal bleeding with passage of clots, so she was referred to our hospital for further management. 

Upon arrival at the ER, the patient was evaluated by the emergency physician and gynecologist and found to have fever spikes of 101°F, tachycardia with a heart rate of 160/min, and very low blood pressure where systolic blood pressure was 70 mmHg, and diastolic blood pressure was undetectable. Intravenous fluids were administered: 30 mL/kg stat as per sepsis protocol, but in this case, we did 1.5 L due to her underlying cardiac condition and possibly pulmonary edema. Norepinephrine was started and then up-titrated, followed by vasopressin, which was added and slowly up-titrated. Intravenous antibiotics such as cefoperazone + sulbactam and IV clindamycin, tranexamic acid, intravenous injection of vitamin K, and intravenous fluids were initiated for better hemostasis control. Subsequently, she was transferred to the medical intensive care unit (ICU) for further management.

Laboratory investigations, as shown in Table 1, revealed a very low hemoglobin level (5.4 g/dL), significantly elevated total leukocyte counts (TLCs) (WBC, 21,000), raised international normalized ratio (INR) (INR, 2.46; PT, 31.8; APTT, 13.7), very high levels of troponin I (7.13 ng/mL), increased urea and creatinine levels, and markedly elevated N-terminal pro-B-type natriuretic peptide (NT-proBNP > 25,000) levels. 2D ECHO imaging showed a mildly dilated LV, global LV hypokinesia, severe LV systolic dysfunction, an ejection fraction of 25%, and an RVSP of 40 mmHg.

**Table 1:** Laboratory values of the patient NT-proBNP, N-terminal pro-B-type natriuretic peptide

Laboratory tests	Patient laboratory values	Reference laboratory values
Hemoglobin	5.4 g/dL	12-15 g/dL
Total leukocyte count	21,000 cells/cu.mm	4000-11,000 cells/cu.mm
Prothrombin time	31.8 seconds	10-13 seconds
International normalized ratio	2.46	0.8-1.1
Activated partial thromboplastin time	13.7 seconds	21-35 seconds
Troponin I	7.13 ng/mL	0-0.04 ng/mL
Blood urea	57 mg/dL	6-24 mg/dL
Creatinine	1.4 mg/dL	0.6-1.1 mg/dL
NT-proBNP	>25,000 pg/mL	<300 pg/mL

The patient was further transfused with six units of fresh frozen plasma and four units of packed red blood cells in the ICU, and the family was counseled about the poor prognosis due to life-threatening complications from ongoing vaginal bleeding.

Further consultation with interventional radiology revealed a ruptured pseudoaneurysm in the left uterine artery on angiogram. Emergency bilateral UAE was performed under local anesthesia to arrest the ongoing bleeding. However, her clinical course was further complicated by severe sepsis and stress cardiomyopathy, leading to septic shock with superimposed cardiogenic shock due to TCM requiring escalation of antibiotics and initiation of non-invasive ventilation (NIV) support.

**Figure 1:**
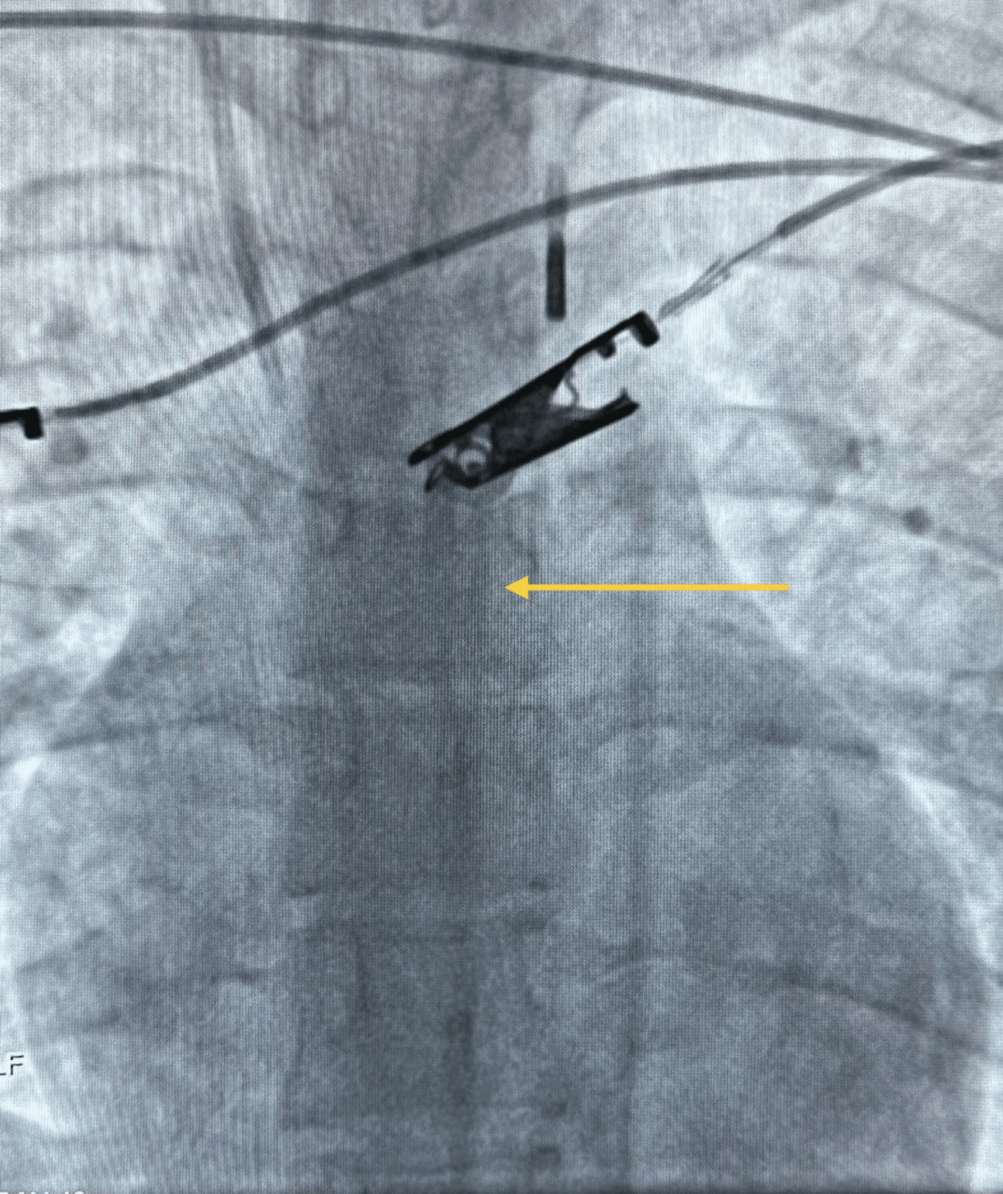
Chest X-ray showing IABP insertion in the patient’s aorta IABP, intra-aortic balloon pump

An angiography done by the cardiologist led to the diagnosis of stress cardiomyopathy with apical ballooning, as seen in Video 1. Consequently, an IABP was inserted for refractory hemodynamics, as shown by the yellow arrow in Figure 1.

**Video 1:** Angiography showing apical ballooning consistent with Takotsubo cardiomyopathy

Initially, the patient had elevated inflammatory markers and fever, indicating an ongoing inflammatory response that was challenging to manage. However, despite these challenges, the patient’s blood pressure improved significantly, reducing the need for vasopressors, gradually weaning off them entirely, and then removing the IABP. These improvements suggest that the underlying condition necessitating these interventions is responding well to treatment.

After discharge, her clinical condition had improved significantly, with hemodynamic stabilization and resolution of febrile spikes. Though she had moderate to severe left ventricular dysfunction on echocardiography, her overall prognosis was favorable.

This case highlights the complex management needed for secondary PPH with associated complications, including hemorrhagic shock, severe anemia, sepsis, and stress cardiomyopathy. A multidisciplinary approach, including obstetricians, cardiologists, interventional radiologists, intensivists, and pulmonologists, played an essential role in optimizing patient outcomes. Timely interventions, such as IABP insertion and UAE, were crucial in controlling hemorrhage and stabilizing the patient’s condition. Further research is warranted to explore optimal management strategies and improve outcomes in similar high-risk obstetric cases.

## Discussion

Management of a severe case of PPH, which is complicated by TCM, is very challenging. A detailed understanding of how both obstetrics and cardiac pathophysiology interact is needed, along with a meticulously formulated multidisciplinary approach. This section inspects this complex teamwork to attain the best results for the patients.

Optimizing hemostasis and minimizing blood loss

Obstetricians usually have a stepwise approach to the first management of PPH. Uterine massage, oxytocin, and methylergonovine are uterotonic medications intended to increase myometrial contractility and decrease bleeding. UAE may be used in case of failure of conservative measures. This is a minimally invasive procedure, where the uterine arteries are catheterized to deliver embolic agents and cause targeted vascular occlusion that will result in the cessation of blood flow. In severe cases, hysterectomy may be performed as a last-resort measure [8]. 

Close collaboration with cardiologists must occur throughout this process. Transfusion of blood products remains essential to PPH treatment; however, TCM requires a prudent approach [9]. The task before cardiologists is to strike and maintain a balance between ensuring adequate circulating blood volume that would avoid hypovolemic shock and avoiding volume overload that could further compromise an already compromised left ventricle [4]. 

Optimizing cardiac function and hemodynamics

Cardiologists play an essential role in diagnosing TCM using transthoracic echocardiography. This non-invasive imaging technique helps identify the left ventricular apical ballooning characteristic of the disease and differentiates it from acute coronary syndrome [10]. To enhance cardiac function, they can use beta-blockers to decrease the adverse effects of catecholamine surges and dobutamine, an inotropic agent designed to increase contractility and cardiac output [11].

Another important aspect is the use of IABP. Cardiologists assist with the percutaneous insertion of the IABP catheter into the femoral artery, followed by its positioning within the descending aorta. This device’s inflated balloon increases coronary flow at the diastole (the phase when the heart relaxes) and decreases left ventricular afterload, hence improving myocardial perfusion and probably averting further deterioration of the heart. Weaning off IABP support should be done slowly based on hemodynamic parameters and echocardiographic assessment of LV recovery [12].

Balancing anesthetic considerations and pain management

Often, anesthetists change their usual approach to these distinctive physiological situations associated with PPH and TCM. The choice of anesthetic drugs should consider possible inotropic effects and hemodynamic stability. While operating, it is crucial to closely monitor vitals like heart rate, blood pressure, and central venous pressure. It is important to maintain track of the volume, either through blood component therapy or through judicious use of crystalloid fluids, to maintain adequate circulating volume yet avoid the volume overload that precipitates heart failure [13].

Pain management strategies are also important. Uncontrolled pain can trigger a stress response that only makes TCM worse. Despite the vasodilation potential and harmful inotropic properties of opioids, they are still commonly used as analgesic agents, but careful titration is necessitated by their side effects [14]. Regional anesthesia can help manage specific areas of pain while minimizing its systemic effect.

Intensive care comprehensive management unit 

Critical care specialists take over when a patient is admitted to the ICU. They offer advanced life support that includes mechanical breathing when respiratory failure emerges due to hemorrhage or heart malfunctioning. Hemodynamic monitoring through arterial and central venous lines allows for real-time preload, afterload, and cardiac output assessment, assisting in fluid resuscitation and administration of vasopressors [13]. 

Working closely with cardiologists guarantees continued treatment of TCM. Serial echocardiograms are performed to monitor left ventricular function to help decide how long inotropic therapy should continue and whether additional therapies might be necessary [15,16]. Meanwhile, blood loss management proceeds by monitoring hemoglobin and hematocrit levels for further guidance on the requirement for extra transfusions of blood products.

Individualized treatment for different patients

A uniform approach to managing severe PPH with TCM is not effective. Decision-making on treatment is guided by a comprehensive review of multiple factors.

Postpartum Hemorrhage Severity

The quantity and speed of blood loss govern how urgent these interventions should be. Unless they are timely, the above complications could become life-threatening [9].

Hemodynamic State

Blood pressures, heart rates, urine outputs, and lactate levels give information on the adequacy of tissue perfusion and the presence or absence of organ dysfunctions.

Pre-existing Morbidity

Other medical illnesses, such as pre-eclampsia or underlying coronary artery disease, can affect available treatments. These may involve medications that could strain already compromised organs.

Treatment Efficacy

The patient’s response to initial intervention (i.e., uterine massage and medications), as well as hemodynamic response to blood product transfusion and IABP support, influence the need for more aggressive measures such as UAE/rescue therapy/inotropic support.

By personalizing the treatment plan based on these intricate considerations, the multidisciplinary team can significantly improve the chances of successful management and minimize the risk of complications linked with both TCM and PPH [17]. This multi-pronged approach, integrating cardiology, obstetrics, and critical care expertise, forms the backbone of successful treatment in these complicated obstetric emergencies. 

The use of an IABP for the management of severe PPH with TCM is assuring, but further research is required to improve treatment guidelines and optimize patient outcomes. For this group of patients, prospective studies should be conducted to assess the efficacy, safety, and cost-effectiveness of IABP versus standard methods, including components like the amount of blood loss, time taken for hemodynamic recovery, and long-term maternal and neonatal outcomes. Standardized protocols and guidelines for IABP in obstetric emergencies with TCM may be needed to enhance care across different healthcare settings. Some of these are determining which patients should have an IABP inserted into their femoral artery, deciding when they should be weaned off the device based on hemodynamic parameters, and ensuring that there is teamwork between obstetricians and cardiac services, improving patient outcomes.

Furthermore, care for severe PPH can be improved through hemodynamic monitoring, pharmacotherapy, interventional techniques, and other means that will further lead to improved maternal morbidity and mortality rates. Interdisciplinary collaboration between health professionals, researchers, and policy experts is required to address the evolving dynamics related to obstetrical emergencies. This collaboration will increase the chances of improving the situation of women around the world. The healthcare system should support these efforts and creative thinking in PPH management in order to improve maternal health outcomes globally and thereby lead to a decrease in the number of maternal deaths worldwide. This, therefore, calls for future research efforts to be focused on investigations of new therapeutic modalities, strengthening existing protocols and healthcare infrastructure, addressing new challenges in pregnant women, and preventing strategies against life-threatening complications like TCM following PPH.

## Conclusions

In conclusion, managing severe PPH requires a comprehensive approach, incorporating conventional and innovative therapies tailored to individual patient needs. This case report highlights the potential utility of IABP in refractory PPH complicated by TCM, underscoring its role in improving maternal hemodynamics and preventing adverse outcomes. Further research is warranted to establish the safety and efficacy of IABP in obstetric emergencies such as refractory PPH, emphasizing the importance of considering novel interventions in managing life-threatening complications of childbirth.
